# Efficacy and safety of clopidogrel versus aspirin monotherapy for secondary prevention in patients with coronary artery disease: a meta-analysis

**DOI:** 10.3389/fcvm.2023.1265983

**Published:** 2023-10-17

**Authors:** Di Liu, Wei Pan Xu, Hang Xu, Lin Zhao, Dao Qun Jin

**Affiliations:** ^1^ Department of Cardiology, Huangshi Central Hospital, Affiliated Hospital of Hubei Polytechnic University, Huang Shi, China; ^2^ School of Medicine, Wuhan University of Science and Technology, Wuhan, China

**Keywords:** coronary artery disease, antiplatelet monotherapy, aspirin, clopidogrel, efficacy, safety

## Abstract

**Background:**

The benefits and risks of aspirin verse clopidogrel monotherapy in patients with coronary artery disease (CAD) remain controversial. This meta-analysis evaluated the efficacy and safety of aspirin verse clopidogrel monotherapy for long-term treatment in patients with CAD.

**Methods:**

Literature was searched in the Pubmed, the Cochrane Library, and the Embase databases until March 2023. The Cochrane Risk of Bias Tool was used to assess the risk of bias in included studies. Data were extracted from the included studies, heterogeneity analysis, and pooled analysis conducted by RevMan 5.3 software.

**Results:**

A total of five trials were included, involving 11, 766 patients with CAD. Compared with the aspirin group, the clopidogrel group was associated with reduced risk of major adverse cardiac and cerebrovascular events (MACCE) [risk ratio (RR) = 0.68, *P* = 0.0007], myocardial infarction (MI, RR = 0.66, *P* = 0.01), stroke (RR = 0.58, *P* = 0.003), and BARC major bleeding (RR = 0.63, *P* = 0.02). There were no significant differences in death from any cause (RR = 1.06, *P* = 0.59) and vascular death (RR = 0.92, *P* = 0.62) between the two groups.

**Conclusions:**

Patients with CAD use clopidogrel could further reduce the risk of MACCE, MI, stroke, and BARC major bleeding, compared with the use of aspirin. This finding supported the use of clopidogrel rather than aspirin in patients with CAD who required long-term antiplatelet monotherapy for preventing ischemic events.

## 1. Introduction

Cardiovascular diseases are the main cause of death and disability worldwide (1). Coronary artery disease (CAD) is the main cause of cardiovascular event, including myocardial infarction, heart failure, and cardiovascular death. Antiplatelet therapy is the most important for preventing the occurrence of adverse cardiovascular events for patients with CAD. Aspirin, the first-line antithrombotic drug, has been preferred use for primary and secondary prophylaxis in populations at high risk for ischemic events for decades (2). However, a clinical trial indicated the primary prevention of aspirin has little or no benefit for vascular adverse events in the healthy elderly population, instead, it increases the risks of bleeding (3). Therefore, the clinical benefit of aspirin in patients with CAD needs to be further examined. Clopidogrel is the adenosine diphosphate-receptor blocker, has been used as an alternative in patients who could not tolerant aspirin or used in combination with aspirin as a dual antiplatelet therapy (DAPT) to provide a stronger platelet inhibitory effect for patients with acute coronary syndrome or undergoing percutaneous coronary intervention (PCI) (4). Interestingly, both our previous meta-analysis and recent clinical guideline have emphasized that early discontinuation of aspirin and continuation of P2Y12 inhibitor monotherapy for some patients after PCI can effectively reduce the risk of ischemic events, and did not increase the risk of bleeding (5, 6). However, controversy remains on the use of aspirin or clopidogrel monotherapy for long-term antiplatelet therapy in patients with CAD. Therefore, we conducted this meta-analysis intent to systematically compared the efficacy and safety of aspirin verse clopidogrel monotherapy for patients with CAD.

## 2. Materials and methods

### 2.1. Search strategy

Literature in English was searched in the Pubmed, the Cochrane Library, and the Embase databases until March 2023. Search terms were used as followed: (1) Aspirin vs. clopidogrel monotherapy and coronary artery disease; (2) Aspirin vs. clopidogrel and percutaneous coronary intervention; (3) Aspirin monotherapy and coronary artery disease; (4) clopidogrel monotherapy and coronary artery disease.

### 2.2. Selection criteria

The irrelevant publications and duplicates were removed by reading the title and abstract, then screened the leftover articles by reading the full text. The clinical trials included in this study had the following characteristics: (1) Comparing the effects of aspirin and clopidogrel in patients with CAD; (2) Outcomes reported in these articles included ischemic events and bleeding events; (3) Published in English. Other studies were excluded because as followed: (1) other types of articles including reviews, meta-analysis, letter, and retrospective analysis; (2) incompatible comparison; (3) no data available.

### 2.3. Data extraction and quality assessment

Two investigators (Dr. Liu and Dr. Xu) independently read these articles and extracted relevant data and assessed the risk of bias. If there is a difference of opinion emerged during this process, the discrepancies were resolved by Mrs. Zhao. The Cochrane Risk of Bias Tool was used to assess the risk of bias in included studies.

### 2.4. Statistical analysis

Data analysis was performed by Review Manager 5.3 software (Cochrane Collaboration, UK). RR with 95% confidence intervals (CI) was used to count the effects of each study. Heterogeneity among studies was assessed using the Q test and *I*^2^ statistics. A cut-off of *I*^2 ^< 50% and *P *> 0.1 indicates low heterogeneity, and the FEM (fixed-effect model) was used to pool analyze the RR value of each study. *I*^2 ^> 50% and *P *< 0.1 indicated significant heterogeneity, the REM (random effect model) was used to pool analysis RR value, and heterogeneity analysis and sensitivity analysis were conducted. To evaluate the consistency of our findings, the sensitivity analysis was by removing every single trial in order from the pooled analysis. *P *< 0.05 were considered a statistically significant difference.

## 3. Results

### 3.1. Identification and selection of study

**Figure 1:**
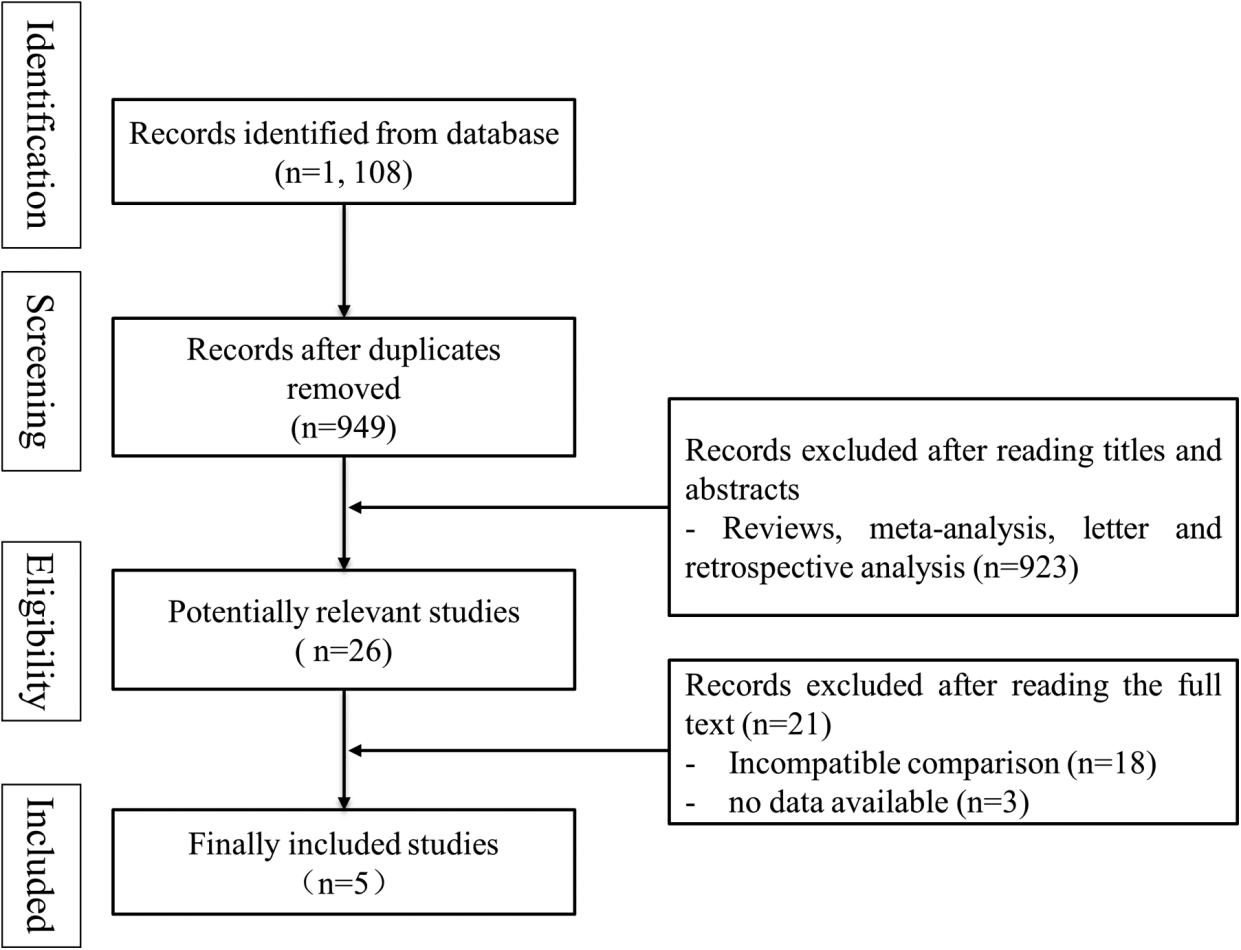
Flow diagram for study selection.

According to the search strategy, 1, 108 records were obtained from databases. 1, 082 records were excluded after reading titles and abstracts (159 articles were duplicates, and 923 articles were not clinical trials). A total of 26 articles were read in full, and finally, five trials were included for meta-analysis (7–11). The process of literature screening was shown in Figure 1.

### 3.2. Basic characteristics of included studies

**Figure 2:**
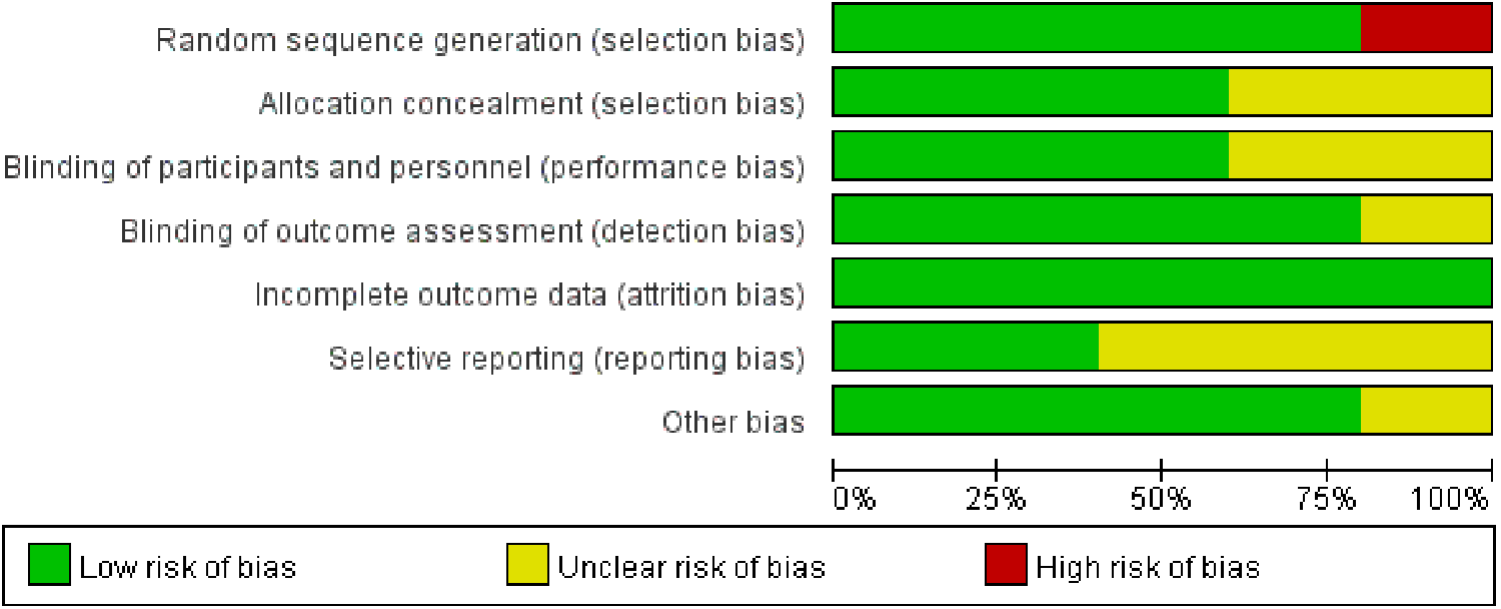
Quality evaluation of the included studies.

Five trials involved a total of 11,766 participants compared the benefits and risks of aspirin vs. clopidogrel in patients with CAD (7–11). The characteristics of these studies were summarized in Table 1. Except for the population of one trial was patients with stable coronary heart disease after coronary artery bypass grafting, the populations of other studies were patients with coronary heart disease after PCI (7). Moreover, the quality of the literature was analyzed and visualized by the Cochrane Risk of Bias Tool (Figure 2).

**Table 1:** Characteristics of included studies.

Variable	Bhatt et al. (7)	Koo et al. (8)	Lemesle et al. (9)	Park et al. (10)	Woodward et al. (11)
Country (*n*, centers)	16 countries (384 centers)	South Korea (37 centers)	France	South Korea (1 centers)	UK (18 centers)
Study design	RCT	RCT	PSM	Observed study	RCT
Study population	Patients with CAD who had a history of cardiac surgery (more than 91% was CABG)	Patients with CAD maintained 6–18 months DAPT underwent PCI with DES	Patients with stable CAD	Patients with stable CAD who had received at least one DES and 12 months DAPT	Patients with CAD who had prior MI
Follow-up	36 months	24 months	24 months	12 months	6 months
Sample size (*n*, A/C)	705/775	2,728/2,710	709/712	2,472/771	90/94
Average age (year, A/C)	63.9/63.3	63.4/63.5	66.5/68.2	62/64	62.4/62.9
Male (%, A/C)	84/83	74.7/74.4	77.9/78.4	73.3/73.9	81/81
Clopidogrel dose	75 mg once daily	75 mg once daily	NA	NA	75 mg once daily
Aspirin dose	325 mg once daily	100 mg once daily	NA	NA	75 mg once daily

A, the aspirin group; C, the clopidogrel group; RCT, randomized controlled trial; PSM, propensity score matching; NA, data not available; CAD, coronary artery disease; DES, drug eluting stent; DAPT, dual antiplatelet therapy; MI, myocardial infarction.

### 3.3. Efficacy outcomes

#### 3.3.1. Major adverse cardiac and cerebrovascular events (MACCE)

**Figure 3:**
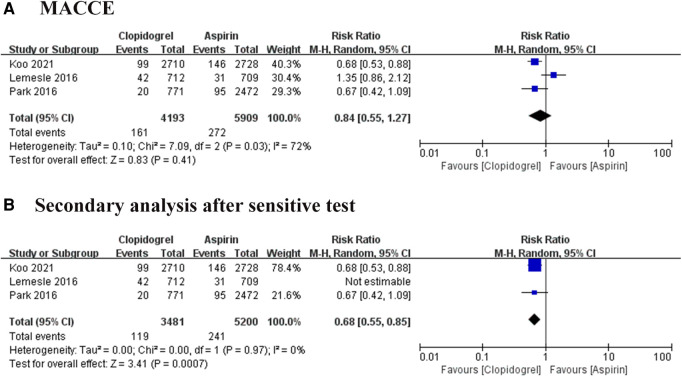
Forest plot of meta-analysis for MACCE. (**A**) Forest plot of meta-analysis for MACCE before heterogeneity analysis; (**B**) forest plot of meta-analysis for MACCE after heterogeneity analysis.

MACCE was defined as a composite of death of all causes, myocardial infarction (MI), stroke, and target vessel revascularization. Three trials reported the MACCE, involving 10, 102 patients (8–10). Heterogeneity among these studies was at a high level (*I*^2 ^= 72%, *P *= 0.03), thus the REM was used to further analysis. The result of pool analysis showed that the aspirin group has a similar risk of MACCE to the clopidogrel group [Risk ratio (RR) = 0.84, 95% CI: 0.55–1.27, *P *= 0.41], as shown in Figure 3A. To eliminate bias due to heterogeneity, we performed a sensitivity analysis. It was found that the result of one study was the main source of heterogeneity (9). After removing this study, the heterogeneity became very low and the pooled results showed clopidogrel could significantly decrease the risk of MACCE compared with the use of aspirin (RR = 0.68, 95% CI: 0.55–0.85, *P *= 0.0007), as shown in Figure 3B. These results suggested that the use of clopidogrel in the prevention of MACCE in patients with CAD is significantly better than the use of aspirin.

#### 3.3.2. Death from any cause

**Figure 4:**
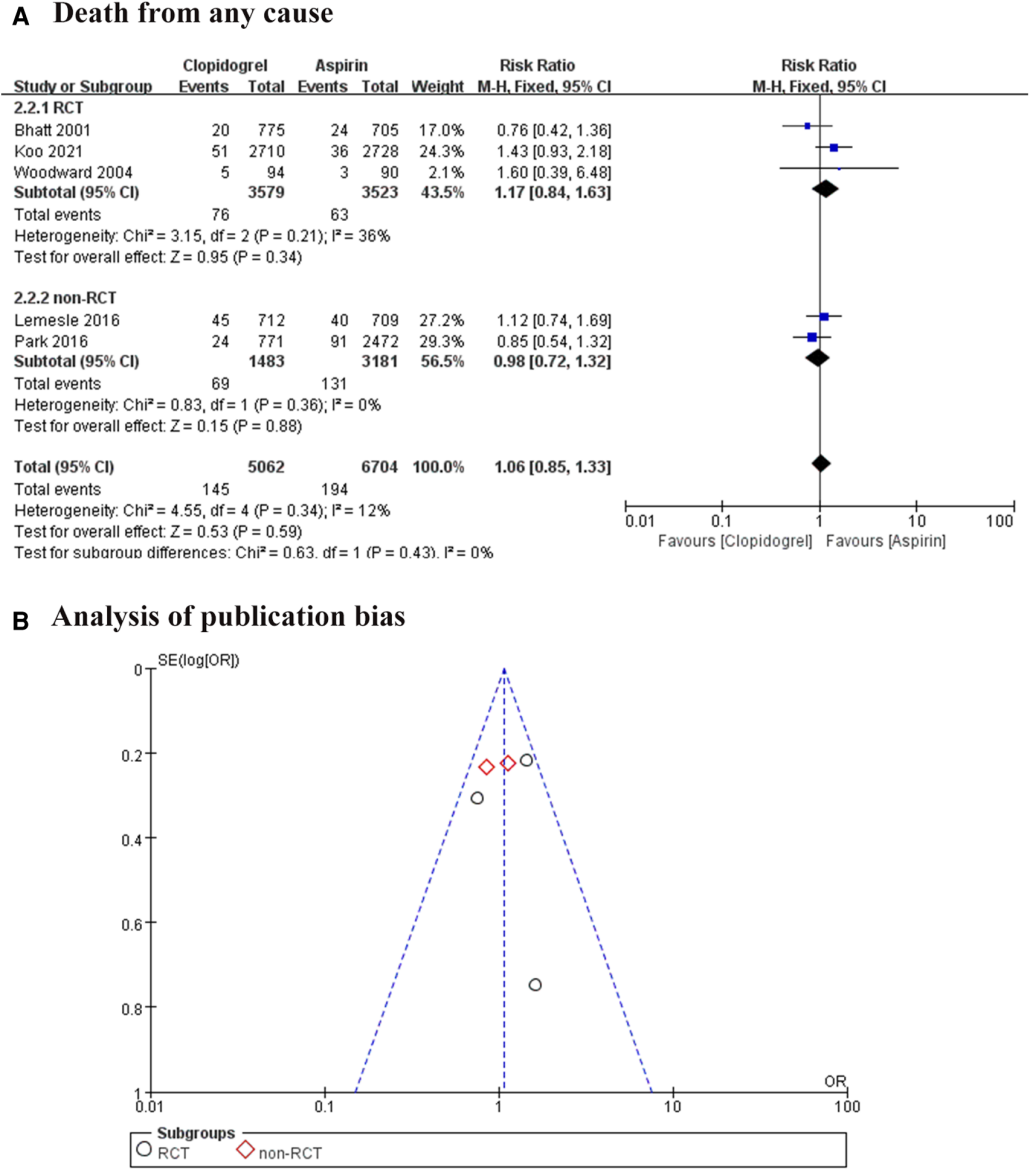
Forest plot and funnel plot of meta-analysis for death from any cause. (**A**) Forest plot of meta-analysis for death from any cause; (**B**) funnel plot of meta-analysis for death from any cause.

All included trials reported the death of any cause, involving 11, 766 patients (7–11). We divided all studies into RCT subgroups or non-RCT subgroups according to the design of each study. Whatever the pooled analysis or subgroup analysis found a low heterogeneity among these trials, and no significant difference in death from any cause between the aspirin group and the clopidogrel group (RR = 1.06, 95% CI: 0.85–1.33, *P *= 0.59; Figure 4A). We also analyzed the publication bias, suggesting the effect calculated by meta-analysis was consistent with the effect of the intervention in included trials (Figure 4B).

#### 3.3.3. MI

**Figure 5:**
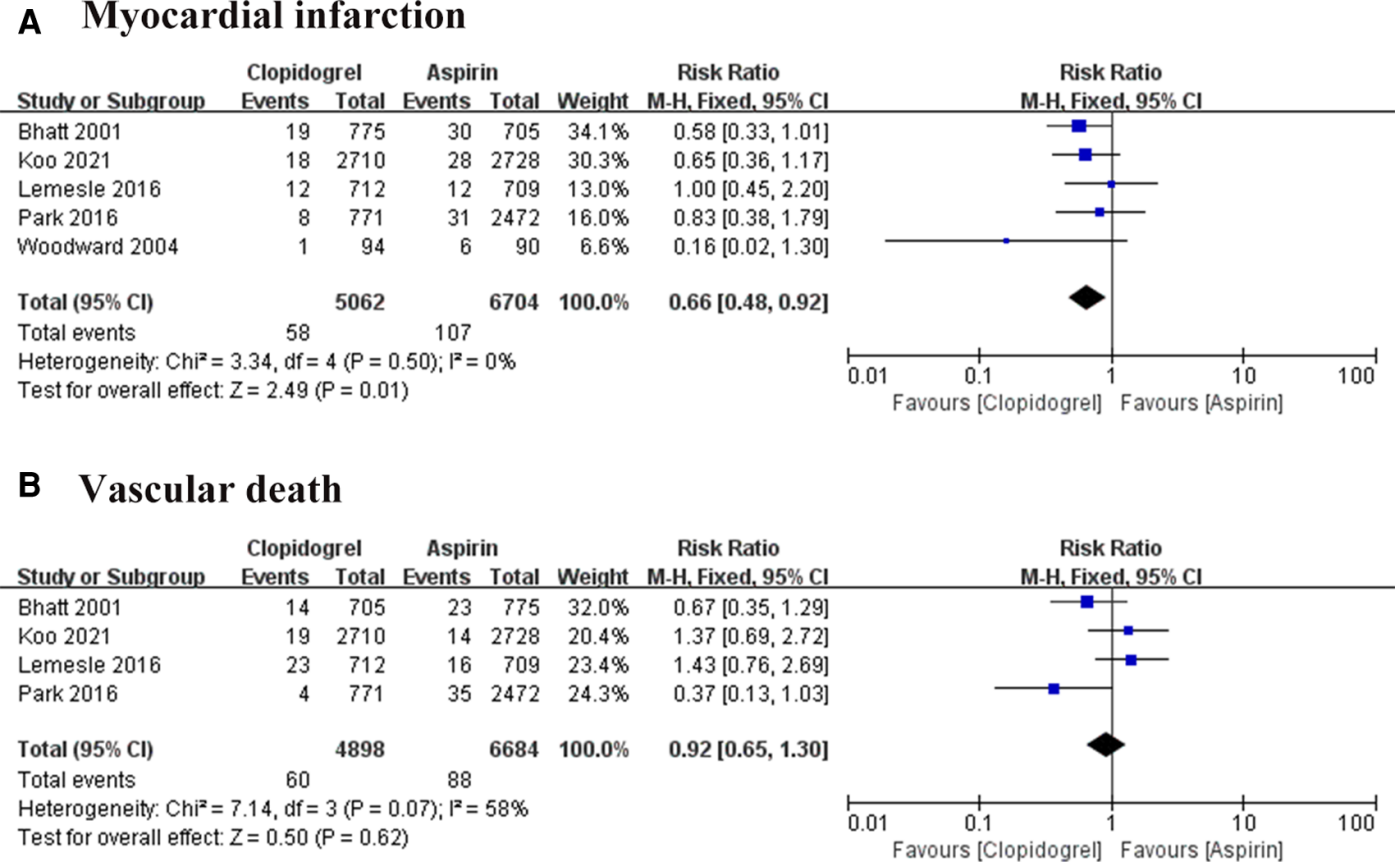
Forest plot of meat-analysis for MI and vascular death. (**A**) Forest plot of meta-analysis for MI; (**B**) forest plot of meta-analysis for vascular death.

Five studies followed 11, 766 patients, 107 times MI occurred in the aspirin group and 58 in the clopidogrel group (7–11). Heterogeneity analysis showed a low heterogeneity among the four trials (*I*^2 ^= 0%, *P *= 0.50), suggesting a consistency of results. The FEM was used for pooled analysis, and the results significantly favor the clopidogrel group (RR = 0.66, 95% CI: 0.48–0.92, *P *= 0.01; Figure 5A).

#### 3.3.4. Vascular death

Cardiac death was reported in four trials (7–10). Heterogeneity among these studies is *I*^2 ^= 58%, *P *= 0.07, suggesting a higher heterogeneity. So, the effect sizes were combined using REM, and results in the forest plot showed there was no difference between the two groups in preventing cardiac death (RR = 0.92, 95% CI: 0.65–1.30, *P *= 0.62; Figure 5B).

#### 3.3.5. Stroke

**Figure 6:**
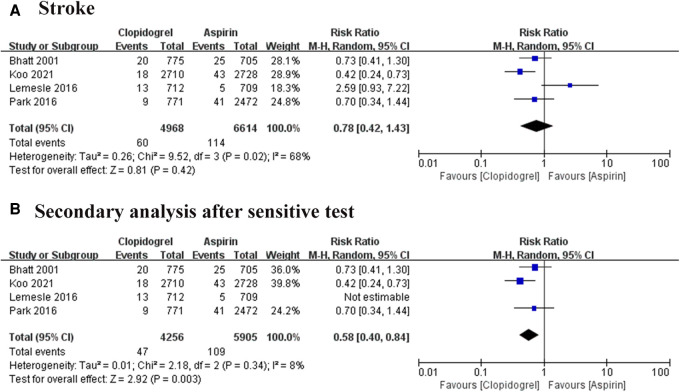
Forest plot of meta-analysis for stroke. (**A**) Forest plot of meta-analysis for stroke before heterogeneity analysis; (**B**) forest plot of meta-analysis for stroke after heterogeneity analysis.

The stroke event was reported in four trials, involving 11, 582 patients (7–10). A high heterogeneity among these studies (*I*^2 ^= 68%, *P *= 0.02), thus the REM was used for combined analysis. The result was shown in Figure 6A, there was no significant difference exist between the two groups, but one study showed a visible heterogeneity (9). A subsequent sensitivity analysis was conducted, confirming this study was the major source of heterogeneity (Figure 6B). After removing this article, the remarkable heterogeneity was eliminated (*I*^2 ^= 8%, *P *= 0.34), and the results of the pooled analysis showed a significant difference between the aspirin group and the clopidogrel group (RR = 0.58, 95% CI: 0.40–0.84, *P *= 0.003).

### 3.4. Safety of outcomes: BARC major bleeding

**Figure 7:**
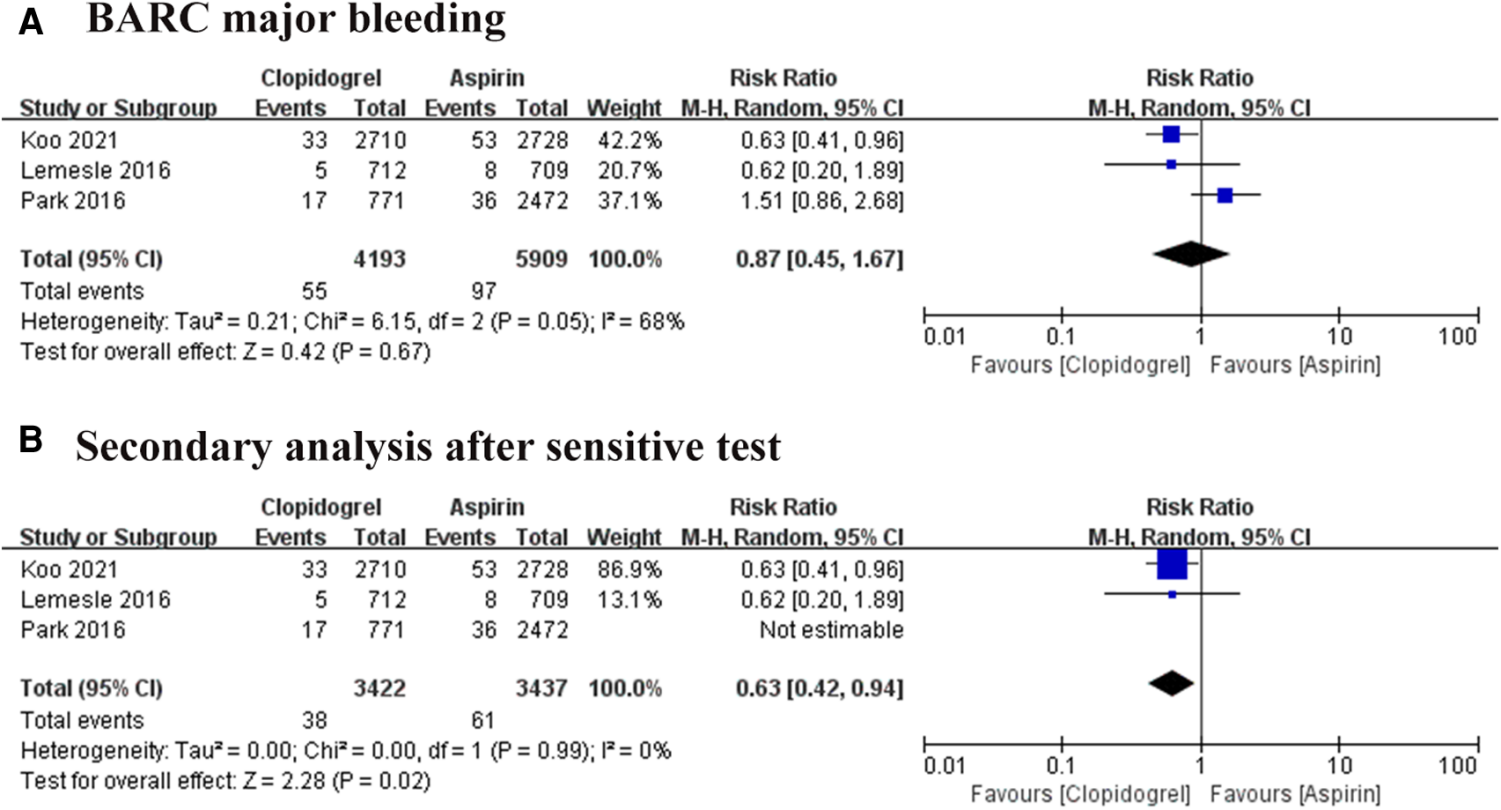
Forest plot of meta-analysis for BARC major bleeding. (**A**) Forest plot of meta-analysis for BARC major bleeding before heterogeneity analysis; (**B**) forest plot of meta-analysis for BARC major bleeding after heterogeneity analysis.

Type 3–5 of BARC bleeding was defined as BARC major bleeding events. Three trials reported this event, involving 10, 102 participants, and a total of 152 positive events were reported (8–10). Heterogeneity among these trials is at a high level (*I*^2 ^= 68%, *P *= 0.05), thus REM was used. The result showed no significant difference between the aspirin group and the clopidogrel group. However, the sensitive analysis found one article (10) is the major source of the heterogeneity, then remove this article. Interestingly, the second analysis showed a distinct result as shown in Figure 7 (RR = 0.63, 95% CI: 0.42–0.94, *P *= 0.02). This result favored the clopidogrel group and suggested aspirin could increase the 60% risk of BARC major bleeding compared with the use of clopidogrel.

## 4. Discussion

In this meta-analysis, we included five studies involving 11, 766 participants with CAD who used aspirin or clopidogrel monotherapy for preventing ischemic events. We compared the efficacy and safety between the aspirin group and the clopidogrel group by heterogeneity analysis, sensitive analysis, and pooled analysis. The efficacy outcomes suggested that the use of clopidogrel is associated with lower risks of MACCE, MI, and stroke in patients with CAD compared with the use of aspirin. The safety outcomes suggested clopidogrel could drop the risk of BARC major bleeding down, compared with the aspirin group. Moreover, using aspirin or clopidogrel had a similar risk of death from any cause and vascular reason in patients with CAD. Given to the benefits of efficacy and safety of clopidogrel for patients with CAD is superior to that of aspirin, we recommend that clopidogrel be preferred as a sustained monotherapy antiplatelet therapy in the clinical scenario.

Guidelines recommend continual treatment with aspirin for preventing ischemic events in patients with CAD. After patients are treated with an adequate course of DAPT, they will be advised to discontinue the P2Y12 receptor inhibitor and continue taking aspirin. However, the ASPREE trial reported an unexpected result that 100 mg of aspirin daily as primary prevention for ischemic events in older healthy adults had a significantly higher risk of hemorrhage but did not reduce a significant risk of MACE (3). People cannot help but doubt the role of aspirin in the primary prevention of CAD in high-risk groups. Recently, the HOST-EXAM trial (8) found that clopidogrel, compared with aspirin, could reduce the risk of ischemic endpoint, all-cause death, BARC major bleeding, and any gastrointestinal complications in patients during the chronic maintenance period after PCI with DES implantation, suggesting that clopidogrel was superior to aspirin for secondary preventing in patients with CAD. Therefore, our study further included related studies on the use of aspirin or clopidogrel in patients with CAD to investigate the efficacy and safety of these two drugs in monotherapy for secondary prevention.

CARPRIE trial was the first RCT to investigate the effects of aspirin and clopidogrel in patients who had a recent ischemic stroke, recent myocardial infarction, or symptomatic peripheral arterial disease (12). The results indicated that long-term administration of clopidogrel for patients at higher ischemic risk is more effective than aspirin in reducing thrombotic composite endpoint. Moreover, antiplatelet therapy is associated with reduced perioperative myocardial infarction and bypass patency in patients undergoing coronary artery bypass grafting. Bhatt and his colleagues compared the use of aspirin or clopidogrel in patients after coronary artery bypass grafting, suggesting clopidogrel can reduce the risk of ischemic composite endpoint, along with a decreased risk of bleeding, compared with aspirin (11). In our study, we found that clopidogrel monotherapy could further reduce the risk of MACCE, MI, and stroke in patients with CAD compared with aspirin, showing the advantage of clopidogrel over aspirin in anti-ischemic events. These results are consistent with the findings of the CAPRIE trial (12) and the HOST-EXAM trial (8). The antiplatelet superiority of clopidogrel to aspirin could be explained by the results of the I-LOVE-MONO trial (13), which showed that clopidogrel could lead to better endothelial function, greater platelet inhibition, and lower coagulation activity. Given these studies and our results, we prefer the use of clopidogrel rather than aspirin in patients requiring long-term antiplatelet monotherapy for preventing ischemic events.

Studies have proven that extending the duration of DAPT failed to offer better anti-ischemic risk than 12-month DAPT, while potentially increasing bleeding events (14). But shortening the duration of DAPT to 1–3 months is associated with a similar ischemic risk to standard DAPT with a significantly lower risk of bleeding (15). It was suggested that more thrombotic adverse events occur within 3 months after PCI, but the bleeding risk is always associated with the use of antiplatelet drugs. In the current era of second-generation drug-eluting stents implantation and intensive statin, we need to pay more attention to the bleeding risks of patients using antiplatelet drugs in the long term. The COMPASS trial (16) reported noteworthy results that gastrointestinal bleeding events during antithrombotic therapy were associated with a nearly 20-fold increased risk of newly diagnosed gastrointestinal cancers (7.4% vs. 0.5%, HR = 20.6) and a 70% increased risk of other cancers (3.8% vs. 3.1%, HR = 1.70). A meta-analysis of 22 trials for aspirin vs. clopidogrel, suggested that aspirin increases the risk of gastrointestinal bleeding but no other bleeding (17). Although the number of studies reporting gastrointestinal bleeding events in our study was too small to be pooled for analysis, results from the real-world trial showed that patients taking aspirin had significantly higher gastrointestinal bleeding events than clopidogrel, which may potentially increase the risk of gastrointestinal tumors. Moreover, in our meta-analysis, the safety outcomes showed that clopidogrel could potentially decrease the BARC major bleeding event, although this difference becomes significant after removing sources of heterogeneity. Interestingly, our results are inconsistent with the safety analysis of Yuan et al. (18), because we included the latest study by Koo et al. (8) and performed a sensitivity analysis. More studies are needed to further compare the bleeding risks of aspirin and clopidogrel monotherapy in patients with CAD.

Our meta-analysis had some limitations. First, due to the few trials available to be included in this study, the heterogeneity of some results was high. Although we did a second analysis after heterogeneity and sensitivity analysis, our results may have a bias to some degree. Second, the population in this study was a mixed cohort of patients with CAD, including patients with stable CAD who did not receive revascularization, those who completed a standard duration of DAPT after PCI, and those who had undergone CABG, thus we could not perform subgroup analyses for this diversity due to our lack of direct access to patients' data. Third, data on minor bleeding and gastrointestinal bleeding were sufficient for pooled analyses because of differences in the observed outcomes of the included studies in this study. Therefore, more eligible studies were required to assess the efficacy and safety of aspirin and clopidogrel for personalized medicine in patients with CAD.

## 5. Conclusions

In conclusion, our results showed that clopidogrel could further reduce the risk of MACCE, MI, stroke, and BARC major bleeding in patients with CAD, compared with the use of aspirin. This finding supported the use of clopidogrel rather than aspirin in patients with CAD who required long-term antiplatelet monotherapy for preventing ischemic events.

## Data Availability

The original contributions presented in the study are included in the article/Supplementary Material, further inquiries can be directed to the corresponding authors.
